# Higher diagnostic accuracy and cost-effectiveness using procalcitonin in the treatment of emergency medicine patients with fever (The HiTEMP study): a multicenter randomized study

**DOI:** 10.1186/s12873-016-0081-6

**Published:** 2016-04-06

**Authors:** Yuri van der Does, Maarten Limper, Stephanie C. E. Schuit, Marten J. Poley, Joost van Rosmalen, Christian Ramakers, Peter Patka, Eric C. M. van Gorp, Pleunie P. M. Rood

**Affiliations:** Department of Emergency Medicine, Erasmus University Medical Center, Rotterdam, The Netherlands; Department of Internal Medicine, Erasmus University Medical Center, Rotterdam, The Netherlands; Institute for Medical Technology Assessment (iMTA), Erasmus University, Rotterdam, The Netherlands; Department of Pediatric Surgery, Sophia Children’s Hospital, Erasmus University Medical Center, Rotterdam, The Netherlands; Department of Biostatistics, Erasmus University Medical Center, Rotterdam, The Netherlands; Department of Clinical Chemistry, Erasmus University Medical Center, Rotterdam, The Netherlands; Department of Viroscience, Erasmus University Medical Center, Rotterdam, The Netherlands

**Keywords:** Procalcitonin, C-Reactive protein, Fever, Emergency medicine, Antibiotics

## Abstract

**Background:**

Fever is a common symptom in the emergency department(ED). Fever can be caused by bacterial infections, which are treated with antibiotics. Often, bacterial infections cannot be ruled out in the ED using standard diagnostics, and empiric antibiotic treatment is started. Procalcitonin(PCT) is a biomarker for bacterial infections, but its role in an undifferentiated ED population remains unclear. We hypothesize that PCT-guided therapy may reduce antibiotics prescription in undifferentiated febrile ED patients. The primary objectives of this study are to determine a) the efficacy, b) the safety of PCT-guided therapy, and c) the accuracy of the biomarker PCT for bacterial infections. The secondary objective is to study the cost-effectiveness of PCT-guided therapy.

**Methods/design:**

This is a multicenter noninferiority randomized controlled trial. All adult ED patients with fever(≥38.2 °C) are randomized between standard care with and without the addition of a PCT level, after written informed consent.

- For efficacy, the reduction of patients receiving antibiotics is calculated, using a superiority analysis: differences between the PCT-guided group and control group are assessed using a Fisher’s exact test, and a multivariable logistic regression analysis to account for the effects of demographic and medical variables on the percentage of febrile patients receiving antibiotics.
- Safety consists of a composite endpoint, defined as mortality, intensive care admission and ED return visit within 14 days. Noninferiority of PCT will be tested using a one-sided 95 % confidence interval for the difference in the composite safety endpoint between the PCT-guided and control groups using a noninferiority margin of 7.5 %.
- Accuracy of PCT and CRP for the diagnosis of bacterial infections will be reported, using the sensitivity, specificity, and the area under the receiver-operating-characteristic curve in the definitive diagnosis of bacterial infections.

The sample size is 550 patients, which was calculated using a power analysis for all primary objectives. Enrollment of patients started in August 2014 and will last 2 years.

**Discussion:**

PCT may offer a more tailor-made treatment to the individual ED patient with fever. Prospective costs analyses will reveal the economic consequences of implementing PCT-guided therapy in the ED.

**This trial is registered in the Dutch trial register:**

NTR4949

## Background

Fever is one of the most common symptoms of patients visiting the emergency department (ED). The etiology of fever is diverse, ranging from infectious diseases to neoplasms and trauma [1]. Specific etiologies of fever, such as severe bacterial infections, have to be treated within one hour after ED presentation with adequate antibiotic therapy, according to the surviving sepsis guidelines [2].

Because time is of the essence in the initiation of therapy, physicians in the ED have a limited time window for diagnosing the etiology of fever. This results in a “better safe than sorry” approach, in which broad-spectrum antibiotics are administered to febrile patients, only based on history and physical examination, and readily available diagnostic entities.

On the other hand, antibiotic resistance is becoming an increasing problem worldwide. Antimicrobial stewardship advocates thoughtful initiation of antibiotic therapy. Thus, in treatment of bacterial infections, both under-treatment and overtreatment are undesirable. Therefore, it is vital to increase the accuracy of diagnostics of febrile illness.

The mainstay of diagnosing the etiology of fever in the ED consists of history, physical examination and laboratory analysis of serum and other bodily fluids, and chest X-ray examinations. Cultures and viral throat swabs are obtained, but are of no use in the ED, because results take several hours to days and treatment has to be started early after ED presentation [2]. Currently, leukocyte count, with or without leukocyte differentiation, and C-reactive protein (CRP) are the laboratory discriminators of choice in the initial approach in the diagnostic process of febrile diseases.

A higher accuracy of ruling in or ruling out bacterial infections using biomarkers may result in more accurate antimicrobial therapy. On the individual patient level, fewer patients would be treated empirically with antibiotics. Adverse events and drug interactions would be reduced. Also, a more accurate diagnosis could save hospital expenses and result in cost reductions. On a population level, antibiotics resistance could be countered. For patient safety, this should obviously be without an added risk of under-treatment.

Procalcitonin (PCT) is a promising biomarker for bacterial infections. PCT is a precursor protein of calcitonin. Unlike calcitonin, which is only produced in the C-cells of the thyroid gland, PCT can be produced ubiquitously throughout the human body. The production of PCT is upregulated by proinflammatory cytokines like interleukin -1 (IL-1), IL-2, IL-6 and tumor necrosis factor alpha, and directly by bacterial endotoxins and lipopolysaccharide. Interferon gamma, a cytokine associated with viral infections, reduces the upregulation of PCT. It has been shown that PCT levels in non-infectious febrile conditions, such as autoimmune diseases or fever caused by malignant disorders stay low, whereas CRP levels often rise significantly [3]. Furthermore, an increase in PCT levels can be monitored within 4 to 6 h after start of infection [4–6]. In comparison, CRP is an acute phase protein synthesized exclusively in the liver. CRP levels increase during inflammatory states, but are not specific for bacterial infections and take more time, 6 to 48 h after start of infection, to be detectable compared to PCT [5, 7]. These characteristics give PCT a theoretical advantage over CRP.

Recently, clinical studies have focused on PCT as a biomarker of bacterial infections, showing better diagnostic properties than commonly used markers such as CRP [3, 8–14]. Studies have shown that PCT-guided antibiotic therapy- i.e. starting or withholding antibiotics, based on the PCT-level - is safe and reduces prescription of antibiotics in several distinct patient groups: savings of more than 23 % in the intensive care unit (ICU) and up to 78 % in the general practice setting [8, 11, 15]. However, evidence of the effectiveness of PCT-guided antibiotic therapy in an undifferentiated ED population is scarce.

The ED is the gateway of hospital healthcare. Patients enter with symptoms and leave with a diagnosis. Either they are admitted, or are sent home. For almost every diagnosis, there are numerous medical tests and interventions available that can be utilized in the ED. However, budgets are limited and should therefore be used in the most efficient manner possible. ED based cost-effectiveness studies are used to estimate the value for money offered by new technologies. These studies serve as a tool for hospital administrators and decision makers who are responsible for prioritizing interventions under economic constraints [16].

Estimated yearly costs associated with antibiotic resistance in Europe are in the range of 1.5 billion euros, comprising extra healthcare costs and productivity losses caused by infections due to antibiotic-resistant bacteria [17]. PCT-guided therapy may contribute to a reduction of these enormous costs. It may lead to a reduction in costs of antibiotics and additional diagnostics tests, such as blood cultures in non-bacterial disease. Costs of hospital admissions could be reduced as admission for observation may become obsolete. As an added effect, there may be a reduction of productivity losses, related to paid and unpaid work. Patients may be able to return to their daily activities faster.

The current evidence on the cost-effectiveness of PCT-guided therapy consists of retrospective hypothetical models. One study analyzed the cost-effectiveness of PCT testing in patients hospitalized for community-acquired pneumonia with a mathematical model [18]. The authors showed that a PCT-guided protocol would cost less and would be more effective than usual care [18]. Recently, a model-based study from the US assessed the economic impact of PCT testing in patients with acute respiratory tract infections. The authors documented that PCT-guided care was cost saving in inpatient, ICU and outpatient settings, mainly due to a reduction in antibiotic costs. Total yearly savings at the US national level were calculated at 1.6 billion dollars [19].

These estimates only included costs of PCT testing and savings on costs of hospitalization, excluding savings from other care consumption and productivity losses, which cannot be estimated at this stage. There is a need to broaden the evidence base to promote the efficient use of PCT-guided therapy in patients with fever presenting at EDs.

The primary objectives of this study are to determine a) the efficacy and b) the safety of PCT-guided therapy, and c) the accuracy of the biomarker PCT for bacterial infections. The secondary objective is to study the cost-effectiveness of PCT-guided therapy.

## Methods and design

The HiTEMP study is designed as a multicenter noninferiority randomized controlled trial on PCT-guided therapy. The study will be performed in both academic and non-academic teaching hospital settings. Patients will be asked for written informed consent. Patients who consent are randomized using an online computer program, to either the standard-of-care diagnostic workup of febrile patients (control group), or the standard-of-care workup with the addition of the diagnostic biomarker PCT.

### Outcome measures

#### Primary outcome measures

- The efficacy of PCT-guided therapy is defined as the percentage of patients who are prescribed antibiotics in the ED.
- The safety outcome measure consists of a composite endpoint of 30 days mortality, intensive care unit (ICU) admission within 30 days, or a return visit to the ED within 14 days.
- The accuracy of the definitive diagnosis is reported for PCT and CRP using the sensitivity, specificity, and the AUC of the diagnosis of bacterial infections. The definitive diagnosis is defined in two ways. 1. As “confirmed bacterial infection”, in which culture – and PCR results that fit the clinical case presentation are used to report presence of bacterial infections. 2. As “suspected bacterial infection”, in which two independent physicians will give their expert opinion on the presence of a bacterial infection using culture results, image modalities and clinical course, in case of discrepancy, a third physician will decide.

### Secondary outcome measures

Secondary outcome measures for cost-effectiveness are hospital treatment costs, related medical consumption during follow-up, costs of absenteeism and reduced productivity while at work.

The adherence to PCT advice is reported, including the percentage of antibiotic prescriptions with low PCT result, and withholding of antibiotics in patients with high PCT result.

The percentage of patients who received antibiotics without suspected bacterial infection is reported.

Outcome measures are reported for the total patient population, and are stratified by source of infection: respiratory tract infections, urinary tract infections, skin and soft tissue infections, central nervous system infections, abdominal infections, non-infectious fever, fever without source, and other causes of fever.

### Inclusion and exclusion criteria

Inclusion criteria: All patients of 18 years and older visiting the ED, with a temperature of ≥ 38.2 °C (100.8 °F) are eligible. Temperature is measured using an ear thermometer, in triage. Eligible patients need to provide written informed consent.

Exclusion criteria: Patients with specific immunocompromised status, defined as neutropenia with absolute neutrophil count less than 0.5×10^9^/L, current chemotherapy, or post-organ transplantation are excluded. Furthermore, pregnant patients, moribund patients and patients with a diagnosis that requires primary surgical intervention, or within 72 h after surgery are excluded. Patients are randomized using a minimization procedure [20]. The factors for minimization are imbalance in randomization result and study site. The randomization result is allocated at patient enrolment by a computer generated algorithm.

### Sample size calculation and statistical analyses

Sample size and power analysis: For the three primary objectives the following analyses are used: a) a superiority analysis for the reduction in the percentage of febrile patients receiving antibiotics. b) a noninferiority analysis for safety, and c) an analysis of the test characteristics (sensitivity and specificity) of the biomarker PCT and CRP. The total sample size calculation is based on a power analysis for each of these three primary outcome measures; the total sample size is the maximum of the required sample sizes for the different primary outcome measures.

- For the superiority analysis of the reduction in the prescription of antibiotics, we assumed that antibiotics are initially prescribed at the ED to 73 % of the patients in the control arm, and that the intervention will reduce this percentage to 53 %, based on a careful estimate using recent literature [14]. To obtain 80 % power to detect a significant difference between the two groups using a two-sided Fisher’s exact test with a significance level of 5 %, the required sample size is 101 patients per group.
- The noninferiority analysis for the safety objective uses a composite endpoint consisting of ICU admittance, 30-days mortality or second ED visit within 14 days. For an accurate sample size calculation, we first analyzed ED data from the Erasmus MC from January 2011 until May 2012. In this data set, the prevalence of the composite safety endpoint was 12.7 % (102 cases out of 809 patients). We used this percentage as the expected rate in the control group. Using a noninferiority margin of 7.5 %, a noninferiority analysis comparing the rates in the control and the intervention groups requires 244 patients in each group to obtain 80 % power with a one-sided alpha of 5 %. To account for 10 % expected dropout, the total sample size must be 550 patients.
- The third sample size calculation is based on a comparison of sensitivity and specificity of PCT and CRP in patients with infection. We used data from a meta-analysis [21, 22] to estimate the accuracy of PCT and CRP for bacterial infection. We conservatively assumed zero correlation between the test results of PCT and CRP for the diagnosis of bacterial infection. For the prevalence of bacterial infections, we used data from our pilot study [3] to obtain a representative estimate of the prevalence of bacterial infections, which led to an estimated prevalence of 68 %. Based on the results of the meta-analysis, the sensitivity of PCT was 0.83 and for CRP 0.73; the specificity was 0.88 for PCT and 0.60 for CRP. Using McNemar’s test, 340 patients with a bacterial infection (340/0.68 = 500 patients in total) are required to obtain 80 % power for detecting a difference in sensitivity between PCT and CRP. Forty-one patients without a bacterial infection (41/(1–0.68) = 129 patients in total) are required to obtain 80 % power for detecting a difference in specificity between PCT and CRP. To ensure sufficient power for all three primary outcome measures, we thus use a sample size of 275 patients per group (550 patients in total).

Statistical analyses for the primary study parameters:

- Antibiotics use. The percentage of patients that are prescribed antibiotics is compared between the PCT-guided group and control group using a Fisher’s exact test. Demographic parameters (age, sex, mortality), medical parameters (medication use, comorbidity, temperature, CRP and PCT measurements), hospital admission related parameters (hospital admittance, hospital length of stay, ICU admittance, ICU length of stay) are compared between the PCT-guided group and control group using Fisher’s exact tests for dichotomous variables, chi-square tests for categorical variables with more than two categories, t-tests for continuous variables that are normally distributed, and Kruskal-Wallis tests for continuous variables that are not normally distributed. A logistic regression analysis will be used to determine the influence of these parameters on the proportion of antibiotics prescriptions in the control group and the PCT-guided therapy group. To select the independent variables in this logistic regression model, we will use a stepwise backward approach in which only the independent variables are retained that have a significant effect using a significance level of 5 %; however, the variable group (PCT-guided group versus control group) will be included in the model irrespective of the results. In the event of missing values in possible confounding data, like intoxications and prescription drugs use, we will use multiple imputation. Both an intention to treat analysis and a per protocol analysis will be performed. Physician adherence to the PCT guidance will be determined.
- Safety. Significant differences between the PCT-guided group and control group in the composite safety endpoint are determined using a one-sided upper 95 % confidence limit for the difference in proportion between the PCT-guided group and the control group. This confidence interval will be calculated using the method of Agresti and Caffo [23]. Noninferiority of the intervention will be established if the 95 % confidence interval excludes 7.5 %, i.e. if PCT-guided prescription of antibiotics does not increase the rate of the composite endpoint by more than 7.5 percentage points. Differences in hospital length of stay will be assessed using the Kruskal-Wallis test.
- Accuracy of PCT and CRP. In all patients, the final diagnosis of the etiology of fever will be determined. Also, it will be determined if there is a bacterial infection. The variables “confirmed bacterial infection” and “suspected bacterial infection” will serve as gold standards for the evaluation of the accuracy of the biomarkers CRP and PCT. These will be determined retrospectively, using culture results and all diagnostic tests available. In all patients, both PCT and CRP levels will be available for statistical analysis. The ability to predict bacterial infection of both PCT and CRP will be evaluated using receiver operating characteristic curves, and the area under the curve will be calculated. In addition, logistic regression will be performed to analyze the effects of PCT and CRP levels on the probability of a bacterial infection. The independent variables in this analysis are age, sex, PCT, CRP, temperature, comorbidity, and other variables that have a *p* < 0.1 for the difference between groups. We will account for possible non-linear effects of age, PCT, CRP and temperature by using appropriate transformations of these variables.

## Statistical analyses for the secondary study parameters

The study will involve an economic evaluation from the societal perspective comparing PCT-guided therapy with usual care. The economic evaluation will use the technique of cost-minimization analysis, which compares two interventions of identical effectiveness to find out which is less costly. Total treatment costs will be compared between the PCT-guided therapy arm and the control arm, including costs for PCT testing (intervention group only), other diagnostic tests, ED visits, antibiotics and other medications, adverse effects of antibiotics, hospital admissions, return visits to the general practitioner (GP) and other related medical consumption. These costs will be taken into account during the one-month follow-up period. Unit prices will be calculated using real economic cost prices or using standard cost-prices for health economic evaluations [24].

Unit prices will be multiplied by the quantities for each resource used, and then summed over the separate types of resource to give a total cost per patient. In addition, differences in labor productivity losses will be evaluated by comparing costs of absence from work (absenteeism) and reduced productivity while at work (presenteeism). Moreover, productivity losses related to unpaid work (e.g., household work, shopping, odd jobs, and voluntary work) will be included. Productivity losses will be evaluated using the Productivity Costs Questionnaire [25]. Mean total costs will be calculated for patients in each treatment group.

### Laboratory examinations

Blood samples will be obtained at inclusion. Samples will be centrifuged (3000 N Relative centrifugal force (Rcf) at room temperature for 5 min). The serum will be measured on the routine analyzer of the clinical chemistry laboratory (Roche Cobas 8000 system, Roche Diagnostics Netherlands). PCT-measurements will be performed by using an electro-chemiluminiscent immunoassay (ECLIA) (Roche diagnostics, Brahms, Henningsdorf, Germany). All samples will be measured and reported without knowledge of the clinical status of the subjects.

### PCT-guided therapy

PCT-guided therapy is defined as the initiation of antibiotics, based on all available diagnostics with the addition of PCT-levels. The PCT results are appraised using a two-point scale, in which bacterial infections are respectively deemed unlikely (PCT < 0.5 μg/L) and likely (PCT ≥ 0.5 μg/L). These cut-off values are used in other trials [26–28].

### Follow-up

One month after inclusion, patients will be contacted by telephone by one of the investigators. Course of the disease, including medicine use, related GP hospital visits (and diagnostics/prescriptions), and labor productivity losses, will be evaluated. Three months after inclusion, one of the investigators will contact patient’s GP in order to evaluate the final outcome of the febrile episode. Patients are allowed to participate only once.

### Adverse events

The composite safety endpoint is continually monitored during the trial. Also, adverse events, not in the composite safety endpoint - death after 30 days, life threatening events or any other important medical event that may jeopardize patients in any way - are monitored by the data safety monitoring board of the study.

### Ethics approval and trial registration

The ethics committee of the Erasmus University Medical Center, in Rotterdam, the Netherlands, approved this trial. It is conducted according to the principles of the Declaration of Helsinki, version March 1, 2013 and in accordance with the Dutch Medical Research Involving Human Subjects Act (Wet Medisch wetenschappelijk Onderzoek met mensen, WMO). The CONSORT statements and its revised extension for reporting noninferiority trials were consulted and taken into account when designing and writing this protocol [29, 30]. The trial has been registered in the Dutch trial registry, NTR4949.

## Discussion

Although observational studies have shown that PCT is more sensitive and specific for bacterial infections in comparison to CRP [14, 31], and prospective trials have investigated the reduction of antibiotics in patients with respiratory complaints [32, 33], the role of PCT in the ED remains unclear. This study is designed to answer the question whether PCT can aid in improving the accuracy of diagnosing bacterial infections in an ED setting. The real-life clinical ED setting differs from a research setting in which patients have very specific symptoms. Consequently, the value of PCT for ED patients without specific complaints has not been determined.

Because of this clinical problem, we used an objective measurement of temperature of 38.2 °C or higher as sole inclusion criterion. As a result, we have a more heterogeneous population compared to other trials [32, 33], allowing us to extrapolate the results to the majority of the adult ED patient population. Furthermore, we will be able to identify cases where physicians disregard the PCT level, and investigate the causes. These are urgent questions in emergency medicine.

The costs of PCT-guided therapy may be of influence in implementation in clinical practice. In the Netherlands alone, 94.2 billion euros was spent on healthcare in 2013, and the costs are rising [34]. Therefore, there is much interest in healthcare initiatives that benefit patients’ health and are not associated with additional costs. Currently, there are no prospective cost-effectiveness studies on PCT-guided therapy. Two economic evaluation studies [18, 19] report that a PCT-guided antibiotic algorithm may reduce costs. However, physician adherence to the PCT-guided therapy protocol could not be accurately estimated, and productivity losses are not analyzed. Therefore, these studies do not provide concrete evidence. In the HiTEMP study, costs are analyzed prospectively to provide a more accurate recommendation.

To reduce the risk of harming patients who receive PCT-guided therapy, we designed a noninferiority study protocol for the primary safety objective. Our study is in its design largely similar to the ProHOSP trial [33]. This ED based noninferiority trial, which investigates the start of antibiotic therapy using PCT in patients with suspected respiratory tract infections, uses a composite endpoint with a noninferiority margin of 7.5 %. Moreover, in the Cochrane review on PCT-guided therapy, no differences in mortality or treatment failure were found [35]. We therefore consider the noninferiority margin of 7.5 % of the composite endpoint ethically acceptable. We use a composite endpoint, consisting of patient centered outcomes. These outcomes, i.e. mortality, ICU admittance <30 days or return to ED within two weeks, are a potential sign of treatment failure.

There are logistical issues that may arise during the course of the study. One of the most important issues may be physician protocol adherence. Physicians need to learn how they should interpret the value of the PCT level, and develop a certain clinical’feel’ towards it. The investigators will facilitate adoption of PCT, by informing every physician prior and during the study. Possibly, a ‘learning curve’ can be seen in the treatment according to PCT guidance. However, the physician motivates every choice of treatment that is not in accordance with the treatment advice based on the PCT result. Moreover, the accuracy of PCT will be calculated using the definitive diagnosis. Physician adherence to PCT guidance will also be investigated.

## Conclusions

The HiTEMP trial addresses critical clinical questions in emergency medicine. PCT may offer a more tailor-made treatment to the individual ED patient with fever. The study will also shed light on the cost consequences of implementing PCT-guided therapy in the ED.

### Prospective

The HiTEMP study is open for inclusion. Results are expected at the end of 2016.

### Ethics approval and consent to participate

This study protocol is approved by the medical ethics committee (Medisch ethische toetsingscommissie, METC) of the Erasmus University Medical Center, in Rotterdam, the Netherlands. Reference number MEC 2013–149.

This protocol does not include patient data, therefore informed consent is not applicable.

## Abbreviations

CRP: C-reactive protein
ECLIA: electro-chemiluminiscent immunoassay
ED: emergency department
GP: general practitioner
ICU: intensive care unit
IL: interleukin
PCT: procalcitonin
Rcf: relative centrifugal force
